# Supplementary material for: MetaRibo-Seq measures translation in microbiomes
Source: Nat Commun. 2020 Jun 29;11:3268. doi: 10.1038/s41467-020-17081-z (PMC7324362; doi:10.1038/s41467-020-17081-z)
Supplement: Supplementary file 10 — Supplementary Data 7 [file 41467_2020_17081_MOESM10_ESM.zip › File2/Confidence_High_Taxonomy/82304_out.krona.html]

Javascript must be enabled to view this page.

members
magnitude
magnitudeUnassigned
count
unassigned
taxon
rank

82304\_out

2

superkingdom
2
2

phylum
1224
2

1236
class
2

2
91347
order

2
543
family

1
genus
570


SRS017191\_contig\_number\_contig-100\_9165.53883
1
species
573

1
genus
544

1

SRS971427\_contig\_number\_30558
545
species
